# The Prevalence of Gastric Ulcer Syndrome in 395 Horses in Jiangyin City, China, Jiangsu Province

**DOI:** 10.3390/ani14243636

**Published:** 2024-12-17

**Authors:** Kairen Zhou, Zhen Dong, Xuzheng Zhou, Bintao Zhai, Bing Li, Jiyu Zhang, Fusheng Cheng

**Affiliations:** 1 Key Laboratory of New Animal Drug Project of Gansu Province, Key Laboratory of Veterinary Pharmaceutical Development, Ministry of Agriculture and Rural Affairs, Lanzhou Institute of Husbandry and Pharmaceutical Sciences, Chinese Academy of Agricultural Sciences (CAAS), Lanzhou 730050, China; kairen2010@163.com (K.Z.); dongzhen2022@stu.hunau.edu.cn (Z.D.); zhxuzheng@163.com (X.Z.); zhaibintao@163.com (B.Z.); pharm-2005bl@126.com (B.L.); 2 National and Local Joint Engineering Research Center for the Creation of Traditional Chinese Medicine Resources and Veterinary Drugs, Hunan Province Key Laboratory of Traditional Chinese Veterinary Medicine, College of Veterinary Medicine, Hunan Agricultural University, Changsha 410128, China

**Keywords:** horse, gastric ulcer syndrome (EGUS), glandular gastropathy (EGGD), squamous gastropathy (ESGD), prevalence

## Abstract

The aim of this study was to determine the prevalence and association of EGUS in horses of different ages, breeds and occupations. Gastroscopies were performed on 395 horses, and gastric ulcers were graded on a scoring system from 0 to 4. The relationship between age, breed, and work, along with the prevalence of gastric ulcers and their influences, were evaluated. The prevalence rate of ulcers in this herd was 78%. Older horses were prone to ulcers in both the glandular and squamous mucosal areas. Across the different jobs surveyed, 60% of the horses had a score of 2 or above. For the horses participating in more intense jobs (group performances, pulling carts, etc.), the number of horses with an ulcer score of 2 or above exceeded 50% of the total number of horses in this job. The prevalence of gastric ulcers was high, and these ulcers were severe in the horses belonging to the examined club, with no association between age or breed and the prevalence of ulcers; however, there was a difference in the location of the ulcers between breeds, as well as differences in the incidence and severity of ulcers depending on work activity. This study provides data reference values for the control and prevention of gastric ulcers in horses in this horse farm.

## Simple Summary

This study describes the prevalence of equine gastric ulcer syndrome (EGUS) in horses depending on their age, breed, and job nature. Gastroscopies performed on 395 horses at a horse farm in Jiangyin City revealed differences in the ulcer site depending on horse age and breed site, as well as differences in ulcer incidence and severity, depending on the nature of their work. The findings provide valuable data for managing and preventing EGUS on this horse farm.

## 1. Introduction

The stomach anatomy of equine animals is mainly divided into two regions, namely the squamous mucosa region and the remaining gland-covered region. The fold margin distinguishes the squamous mucosa from the glandular mucosa. Ulcers occurring in these two areas are collectively referred to as equine gastric ulcer syndrome (EGUS), gastric ulcers occurring in the squamous mucosa area are referred to as equine squamous gastric disease (ESGD), and gastric ulcers occurring in the remaining glandular-covered area are referred to as equine glandular gastric disease (EGGD) [1,2,3,4]. EGUS is prevalent across horse populations that are engaged in various activities, including horse racing, show jumping, dressage, and endurance racing, with a particularly high prevalence in high-intensity horse racing [5,6,7]. Stomach ulcers can appear in each position of the stomach, especially on the fold edge; here, the squamous mucosa are more serious and more common [8]. Gastric ulcers are caused by many factors, such as physiological factors such as excessive exposure to gastric acid (hydrochloric acid), and environmental factors including diet, management, and stress, etc. In addition, stomach microorganisms also play a key role in the development of gastric diseases [9,10,11]. Although the cause of ESGD is well established, the specific causative factors of EGGD are less clear, highlighting the need for additional research in this area [12,13,14]. Moreover, a large number of statistics show that horses of any age may develop stomach erosion and gastric ulcers under different breeding and environmental conditions [15,16,17].

In recent years, most international studies have focused on diseases associated with horse racing [18]. Although there are reports on gastric ulcers and their treatment [19,20], most of the investigations focus on independent analysis reports of EGGD or ESGD, with few comparative analyses. Gastroscopy is considered the ‘gold standard’ for the diagnosis of gastric ulcers in horses due to the lack of clear clinical signs. The definitive diagnosis of gastric ulcers in horses can only be made by observing the lesions in the stomach. In addition, diagnostic scores for ulcer severity vary, and lack consistency and relevance; moreover, there is no clear relationship between the conversion of different scoring systems [21,22]. This study will use the “0–4” system proposed by the EGUS Committee and the European EGUS Consensus to grade gastric ulcer lesions [2,12]. It aims to analyze the prevalence of gastric ulcers in the equine performance population in Jiangsu Province, China, and compare the associated factors of EGGD and ESGD. The study aims to provide a reference for the prevention of gastric ulcers in horses.

## 2. Materials and Methods

### 2.1. Animals

The test horses were obtained from the Jiangsu Provincial Equestrian Club. The club is located in Xinqiao Town, Jiangyin City, Jiangsu Province, China (longitude E120°50′, latitude N31°80′). The study was approved by the Animal Ethics Committee of the Lanzhou Institute of Animal Husbandry and Pharmaceutical Sciences of Chinese Academy of Agricultural Sciences. The club has 420 registered horses, all castrated males, aged between 3 and 22 years, with an average age of 13 years and a median age of 13 years; all horses weigh between 200 and 650 kg, and participate in various forms of activity (including pulling carriages for people for 6 h a day, riding for people for 6 h a day, participating in dressage once a week for 50 min, watching for 6 h a day, etc.). Gastric ulcers in horses are challenging to diagnose due to the lack of pathological clinical signs and differences in differentiation from clinical signs [23]. As a result, EGUS cannot be definitively diagnosed, and appropriate medical treatment cannot be administered. A total of 395 horses from this population were examined for the study; the remaining horses were not examined for other reasons (surgical recuperation, outings, etc.), and were not included in the trial. Horses are kept in pens; they can see each other and venture out to participate in related activities every day.

### 2.2. Data Collection

Horse information was collected from club management, including breed/type, age, weight, life stage (young, breeding, working, or retired/not working), and information on workload, management, and nutrition, as well as recent use of NSAIDs and presence of lameness, colic, previously diagnosed EGUS, or recurrent respiratory disease within the previous 6 months. Group trials and statistical analyses were performed on horses according to activity, breed, and age. Horses were fed with self-made feed (including barley flakes, oat flakes, bran, concentrate, soybean oil, etc., containing grain 220 g/100 kg), 7 times a day, at a feeding interval of 3–4 h, then injected with the equine influenza vaccine, tetanus vaccine, and herpes vaccine, which were orally administered with ivermectin anthelmintic, etc. Horses were fed under the same feeding conditions before the test, including feeding times and feed, etc. Horses were housed in standard stables of 3 m × 4 m or 4 m × 4 m, with 30–40 cm of bedding (straw and hay) covering the floor.

### 2.3. Stomach Endoscopy

**Figure 1:**
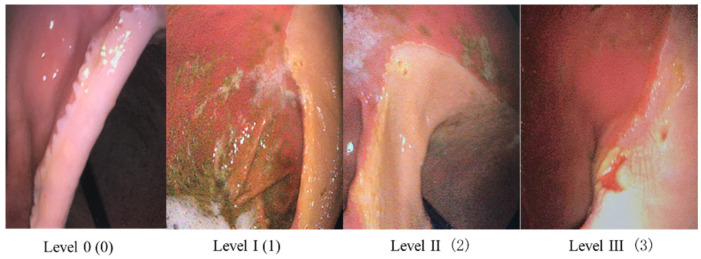
Gastroscopic manifestations of gastric ulcers of different degrees in horses (score 0–3).

Horses were fasted for 10–12 h prior to endoscopy, during which time water was allowed. Each horse was sedated with detomidine hydrochloride (10–15 µg/kg, i.v.) and butorphanol (15–20 µg/kg, i.v.). Before the study, the stomach wall was exposed by infusing air into the stomach through a gastric cannula; this was examined for the presence of gastric ulcers using a 350 cm visual endoscope. The endoscope was coated with a water-based lubricant gel to facilitate passage through the esophagus. The assessment and scoring of ulcers were performed by the same veterinarian. If food consumption interfered with endoscopic observation, large volumes of carbonated water were administered to facilitate gastric emptying. After gastroscopy, the horse was returned to the barn for a 1 h observation period, during which time it was provided with hay and water. After gastroscopy, the horse was returned to the stable for an observation period of one hour before being allowed to eat and drink. The ulcer sites were recorded as squamous mucosa, glandular mucosa, or both. The 0–4 scoring system proposed by EGUS committee and European EGUS consensus was used to grade gastric ulcer lesions (Table 1). Examples of pathologies with scores 0–3 are shown in Figure 1. Because the most severe lesion grade was not found on examination, a score of 4 is not shown in the example picture. The entire evaluation process was evaluated simultaneously by the competent veterinarian of the test horse farm (familiar with all horses) and the competent veterinarian of another horse farm (unknown to any horse on the farm), and the results were determined statistically.

### 2.4. Statistical Evaluations

To investigate the potential effect of different activities, breeds, and ages on the incidence of EGUS in horses, descriptive statistical methods were used to calculate the proportion of horses with gastric ulcers based on their activity, breed, and age. SPSS Statistics 23.0 (IBM Corporation, Armonk, NY, USA) was used for statistical analysis of the data. One-way ANOVA was used to compare data between groups, and post hoc tests were performed using LSD and Games–Howell methods. The proportionate data between groups were analyzed using chi-square tests. The significance level of all tests was 95% (*p* < 0.05).

## 3. Results

The squamous mucosa and glandular sites of all horses tested were thoroughly examined, and no parasites were observed during the examination. Among the 395 examined horses, there were eight different breeds, and horses engaged in seven types of work. Overall, 308 (78.0%) had clinically visible ulcers, of which 123 (40%) were classified as 1, 180 (58.4%) as 2, and 5 (1.6%) as 3. Of these, 29.2% (90/308) developed EGGD. The proportion of horses with a score of 1 was higher at 53.7%, while horses with a score of 2 accounted for 11.7% and horses with a score of 3 for three cases. ESGD occurred in 24.7% (76/308) of cases, and there were no cases of score 3 ESGD. Horses with a score of 1 accounted for 28.4% and those with a score of 2 for 22.8% of cases. Overall, 46.1% (142/308) of the horses had ulcer lesions in both the glandular and squamous mucosa, which was significantly higher than in either the glandular or squamous mucosa alone (*p* = 0.035). The proportion of cases with a score of 2 (65.5%) was significantly higher than that of cases with a score of 1 (17.9%) or a score of 3 (two cases). There were no cases with a score of 2 or above in horses used for children’s riding events, and the number of horses with a score of 2 was significantly higher than that of horses with a score of 2 in other work events (*p* = 0.022); in particular, five cases of horses used in obstacle events with a score of 3 in were recorded (see Table 2 and Table 3).

Lesions located in both the glandular and squamous mucosa were more common than those located in either region alone in horses from three different age groups. Older horses had a higher incidence of gastric ulcers. There was a significant difference between the site of ulcer occurrence and different age groups (*p* = 0.000). Of the 41 horses over 19 years of age with gastric ulcers, none were found to have only glandular ulcers (Table 2).

Frisian, Orlov, and Welsh ponies had an incidence of less than 80%, while the other five types of horses had an incidence of more than 80%. Hanover horses had an incidence of 94.7%. Ulcers occurred only in the glandular or non-glandular parts of Welsh ponies and American horses, whereas ulcers occurred only in the glandular parts of five Welsh ponies. The incidence of EGGD was higher than that of ESGD in Quarter Horses, Hanoverian horses, Mongolian horses, Orlov horses, and Welsh ponies. No cases of ESGD alone were found in Quarter Horses, Orlov horses, and Welsh ponies. The incidence of ESGD was higher in American, Andalusian, and Frisian horses than that of EGGD, with Andalusian horses showing particularly high rates (Table 2).

There was a significant difference between the intensity of work and the location of gastric ulcers (*p* = 0.000). Almost 46.1% of the horses had ulcer lesions in the glandular and squamous areas of the stomach. The prevalence of ESGD was higher in horses used for group work, driving, and viewing than in those used for individual work, and the prevalence of ESGD was lower in other working horses than in esophageal EGGD. No cases of ESGD were found exclusively in show jumping and children’s riding horses, nor were there any cases of both glandular and non-glandular disease in horses used for viewing. Overall, 39.9%, 58.5%, and 1.6% of horses scored 1, 2, and 3, respectively. More than 50% of the horses scored 2 or above for adult riding, group performance, cart pulling, and steeplechase (see Table 2 and Table 3).

## 4. Discussion

The aim of this study was to provide equine clubs with a detailed understanding of the prevalence of gastric ulcers in horses and to analyze the possible relationship between the occurrence of gastric ulcers and the age, breed, and daily work of the horses. In equine gastric ulcer syndrome, glandular mucosal lesions result from factors that affect protective mechanisms, whereas squamous mucosal lesions are primarily caused by ineffective protective mechanisms. Therefore, the presence of lesions depends on the type, intensity, and duration of exposure to factors that predispose the stomach to ulcers [24]. Most of the horses did not have obvious clinical symptoms during the trial, but the club horse breeders informed us that six horses had periodic abdominal discomfort and experienced decreased physical condition. Although abdominal discomfort, decreased appetite, decreased body condition, rough coat, and especially decreased foal vitality, recumbency, and teeth grinding can be clinical signs of GI ulcers in horses [25], not all horses show signs of EGUS on gastroscopy. Therefore, it is not reliable to diagnose EGUS in horses based on clinical signs alone [1,20,23]. All results also merely indicate that EGUS is more prevalent in this horse farm; they do not imply that EGUS incidence is age-related, but merely indicate that EGUS prevalence is higher among older horses on this horse farm.

EGUS can occur at any stage of a horse’s life, including lactation, active career, and resting periods, all of which can lead to varying degrees of impaired physical development and shortened career longevity [26]. Prevalence rates in Thoroughbred horses have been reported to be between 66 and 93%, increasing to 80–100% with continued racing [20]. The prevalence of EGUS in the club’s horses was 78%, with 60% of horses being graded at 2 and above. The prevalence and disease burden were comparable to or worse than those of Thoroughbreds that compete frequently.

EGGD and ESGD have distinct pathogenic factors and pathogenesis, resulting in differences in their incidence [1,12,27]. The incidence of EGGD in the club horses was higher than that of ESGD; these findings are consistent with literature reports, but lower than the 35–72% incidence rates reported in previous studies [11,12,19,28,29]. Although the scoring system has some limitations in assessing the severity of ulcers, it may, to some extent, reflect the health status of the horse population [21]. The number of EGGD cases with grade 1 ulcers was higher than those with ESGD, but lower than those with ESGD. Meanwhile, the ulcer scores of horses with grade 1 and 3 ulcers in the glandular and squamous mucosa were significantly higher, indicating that the horses in this group suffered from severe gastric ulcers.

It has been reported that the severity of gastric ulcers can be judged according to age, i.e., gastric ulcers in older horses may be more severe than those in younger horses [30]. Gastric ulcers in horses are caused by gastric acid when mucosal defense mechanisms are impaired. The main factor in their development is impaired mucosal defense mechanisms [12], which may also be related to inflammatory bowel disease, behavior, and stress. Inappropriate use of NSAIDs can also lead to EGGD [27,31,32]. The incidence of EGUS ranged from 71.0% to 75.6% in different age groups. Older horses were more likely to have both ESGD and EGGD [1], and were also more likely to have severe EGUS [3]. In contrast, foals were more likely to develop glandular ulcers [33]. This phenomenon is not significant in the population studied. In horses over 19 years of age, ulcers are found only in the squamous areas or in both the glandular and squamous areas. This may be related to the increased tolerance of the gastric mucosa in older horses [33]. Therefore, the severity of EGUS in horses is often judged by age alone, but regular comprehensive endoscopic examination is still a reasonable way to determine the presence and severity of EGUS. In addition, any stimulus that has an effect on animal physiology, i.e., stress, should be examined [34,35]. In this study, all horses were led and gently restrained by their familiar breeders in order to reduce the impact of stress on the results of the study. Early studies of EGUS focused primarily on Thoroughbred [29], endurance [7,36], and recreational [28] horses, with a lack of targeted EGUS studies on various breeds of horses. Factors such as breed, personality, and whether a horse lives in a group can cause varying stress levels for the same event [37]. The horse breeds chosen for this study have a lengthy history of hybridization and stable genes. Quarter horses, Hanoverian horses, Mongolian horses, Orlov trotters, and Welsh ponies were found to be more susceptible to EGGD, whereas Andalusian horses are more prone to ESGD. It was indicated that horse breed and genetic factors might be among the influences on gastric ulcers, especially in areas where they are more likely to occur.

Work significantly affects gastric ulcers in horses, particularly horses frequently used for intense activity, which are more likely to develop ESGD (nearly 90%) [5,7]. Irregular and intense activity also contributes to high scores of gastric ulcers [1,7]. Among the club horses, active horses had higher gastric ulcer scores compared to less-active horses, particularly those involved in group shows, adult rides, and carts. The prevalence of EGGD was higher than the prevalence of ESGD in horses commonly used in obstacle racing. Horses with ESGD who participated in group shows, adult rides, and steeplechase events had higher gastric ulcer scores than EGGD scores. Regularly active and more docile horses had a lower prevalence of ulcers and lower severity scores. The results indicate that the daily activities of horses were one factor influencing the occurrence of gastric ulcers, and the severity of gastric ulcers differed depending on the horses’ activities. The greater the frequency and intensity of the work activity, the more severe the ESGD.

## 5. Conclusions

The prevalence of gastric ulcers in horses from equestrian clubs in Jiangsu Province is significantly high and concerning. This necessitates intensive treatment and ongoing attention from club veterinarians to ensure the health and welfare of the horses. Despite its limitations, this survey suggests that age and breed are not appropriate factors in the management of gastric ulcers in horses. Instead, the incidence of gastric ulcers in more heavily worked horses should be of greater concern to trainers and vets. This investigation only examines the prevalence of horse gastric ulcers in this horse farm, which has reference value for the prevention and management of horse gastric ulcers in this horse farm, and provides a data reference for the investigation of pathogenic factors of gastric ulcers in horses.

## Figures and Tables

**Table 1:** Description of scoring system.

Lesion Number Score	Description
0	No lesions
1	1–2 localized lesions
2	3–5 localized lesions
3	6–10 lesions
4	>10 lesions or diffuse (or very large lesions)

**Table 2:** Location and incidence of gastric ulcers in horses of different ages, breeds, and working conditions.

Category	Group	Location				Total	Prevalence Rate			
		EGGD	ESGD	BOTH	HEALTH		EGUS%	EGGD%	ESGD%	BOTH%
Age (years)	Age ≤ 12	49	31	42	32	154	79.2%	31.8%	20.1%	27.3%
	13 ≤ Age ≤ 18	41	30	84	45	200	77.5%	20.5%	15.0%	42.0%
	Age ≥ 19	0	15	16	10	41	75.6%	0	36.6%	39.0%
Type of sort	Quarter	10	0	15	4	29	86.2%	34.5%	0	51.7%
	American	20	25	0	10	55	81.8%	36.4%	45.4%	0
	Andalusia	5	35	20	14	74	81.1%	6.8%	47.3%	27.0%
	Hanover	25	4	42	4	75	94.7%	33.3%	5.4%	56.0%
	Mongolian	15	4	30	8	57	86.0%	26.4%	7.0%	52.6%
	Frisian	5	8	30	34	77	55.8%	6.4%	10.4%	39.0%
	Orlov	5	0	5	5	15	66.7%	33.3%	0	33.4%
	Welsh pony	5	0	0	8	13	38.5%	38.5%	0	0
Type of work	Dressage	9	0	15	4	28	85.7%	32.1%	0	53.6%
	Adults mount	12	4	24	14	54	74.0%	22.2%	7.4%	44.4%
	Children mount	20	0	12	10	42	76.0%	47.6%	0	28.6%
	Group performance	4	30	18	15	67	77.6%	6.0%	44.8%	26.8%
	Drive	9	18	45	16	88	81.8%	10.2%	20.5%	51.1%
	View and admire	16	20	0	20	56	64.3%	28.6%	35.7%	0
	Steeplechase	20	4	28	8	60	86.7%	33.3%	6.7%	46.7%

**Table 3:** Gastric ulcer scores of horses involved in different jobs.

Type of Work	Lesion Number Scores					1			2			3		
	0	1	2	3	4	EGGD	ESGD	BOTH	EGGD	ESGD	BOTH	EGGD	ESGD	BOTH
Dressage	4	9	15	0	0	4	0	5	5	0	10	0	0	0
Adults mount	14	10	30	0	0	6	3	1	6	1	23	0	0	0
Children mount	10	32	0	0	0	20	0	12	0	0	0	0	0	0
Group performance	15	12	40	0	0	4	7	1	0	23	17	0	0	0
Drive	16	24	48	0	0	7	15	2	2	3	43	0	0	0
View and admire	20	16	20	0	0	10	6	0	6	14	0	0	0	0
Steeplechase	8	20	27	5	0	15	4	1	2	0	25	3	0	2

## Data Availability

The original contributions presented in the study are included in the article, further inquiries can be directed to the corresponding authors.
